# Characteristics and Mechanism of Cu Films Fabricated at Room Temperature by Aerosol Deposition

**DOI:** 10.1186/s11671-016-1378-9

**Published:** 2016-03-24

**Authors:** Dong-Won Lee, Oh-Yun Kwon, Won-Ju Cho, Jun-Kwang Song, Yong-Nam Kim

**Affiliations:** Materials & Components Technology Center, Korea Testing Laboratory, 87 Digitalro 26-gil, Guro-gu, Seoul 152-718 Republic of Korea; The College of Information and Communication Engineering, Sungkyunkwan University, Cheoncheon-dong, Jangan-gu, Suwon 440-746 Republic of Korea; Department of Electronic Materials Engineering, Kwangwoon University, 447-1, Wolgye-dong, Nowon-gu, Seoul 139-701 Republic of Korea

**Keywords:** Aerosol deposition, Cu films, Resistivity, Copper oxides, Mechanical interlocking

## Abstract

We were successful in growing a dense Cu film on Al_2_O_3_ substrates at room temperature using an aerosol deposition (AD) method. The characteristics of Cu films were investigated through electrical resistivity and X-ray photoelectron spectroscopy (XPS). The resistivity of Cu films was low (9.2–12.5 μΩ cm), but it was five to seven times higher than that of bulk copper. The deterioration of the resistivity indicates that a Cu_2_O phase with CuO occurs due to a particle-to-particle collision. Moreover, the growth of Cu films was investigated by observing their microstructures. At the initial stage in the AD process, the impacted particles were flattened and deformed on a rough Al_2_O_3_ substrate. The continuous collision of impacted particles leads to the densification of deposited coating layers due to the plastic deformation of particles. The bonding between the Cu particles and the rough Al_2_O_3_ substrate was explained in terms of the adhesive properties on the surface roughness of Al_2_O_3_ substrates. It was revealed that the roughness of substrates was considerably associated with the mechanical interlocking between Cu particles and rough Al_2_O_3_ substrate.

## Background

The rapid growth of wireless communications as well as the use of ubiquitous technology has led to a dramatic increase of interest in the design and fabrication of miniaturized RF/microwave devices [1]. In particular, planar devices such as patch antennas and planar band-pass filters have attracted attention for their high frequency integrated circuits [2–4]. Therefore, the study of metallic materials and metallization processes is necessary to meet the strong need for planar device technology. Copper is a desirable material for connecting circuit elements of integrated circuits with submicron features because of their low resistivity, high thermal conductivity, and low coefficient of resistance [5, 6]. As techniques for Cu deposition, electroless deposition, electroplating, and chemical vapor deposition have usually been used as metallization processes. However, these processes have some environmental problems, such as the toxic wastewater and chemicals generated from the manufacturing and rinsing processes [7–10]. To solve these problems, an environmentally friendly and dry metallization process with no chemical solutions is required.

The aerosol deposition (AD) process as an environmentally friendly, simple, and dry metallization process was recently proposed as an alternative process for electrolyte and electroplating. Moreover, studies for RF devices such as capacitors and filters have been reported because dense ceramic films can be deposited at room temperature using AD process [11–16]. AD is the powder spray-coating method of using a thin/thick film under low vacuum conditions with micron-sized particles [17–20]. The fine particles are ejected through the nozzle and collide with the substrate with high kinetic energy, forming a dense metal film without external heating [21, 22].

In this study, we showed that dense and thick Cu films can be fabricated at room temperature by AD, and the properties of Cu films were investigated in terms of electronic properties and the characterization of the chemical state of copper in the form of Cu and copper oxides (Cu_2_O and CuO) distributed on the surface of two different oxide supports. Moreover, the growth process of Cu films was examined by observing the initial growth stages of Cu films deposited on Al_2_O_3_ substrates. The adhesion strength of grown Cu films on rough Al_2_O_3_ substrates was verified from a tensile pull-off test. To explain the correlation between the adhesion and the mechanical interlocking between Cu films and Al_2_O_3_ substrates, the adhesive strength was analyzed depending on the surface roughness of the Al_2_O_3_ substrates.

## Methods

Cu films were fabricated on the Al_2_O_3_ substrates using an AD method, using Cu powder with a primary particle size of ~2 μm (Nippon Atomized Metal Powders co., Ltd., Japan). The prepared powder was mixed with a carrier gas, and an aerosol was then formed in the aerosol chamber. The aerosol flow was transported through a tube to a slit nozzle (10 mm × 0.04 mm) in a deposition chamber, which was evacuated using a rotary pump with a mechanical booster. The aerosol jet was ejected from the slit nozzle due to the differential pressure between the aerosol chamber and the deposition chamber. Helium gas was used as a carrier gas at a flow rate of 8 L/min. The details of the equipment for the AD process can be found elsewhere [18]. The accelerated Cu particles collided with the prepared Al_2_O_3_ substrates, and Cu films then grew at room temperature.

To observe the growth of the aerosol-deposited Cu films from the initial growth stages, the substrates were scanned in steps. By controlling the concentration of the aerosol, individual Cu-coating layers form on the Al_2_O_3_ substrates for a scanning number from 2 scans to 20 scans. As the number of scans increased, the formation of Cu-coating layers was observed using field-emission scanning electron microscopy (FE-SEM; S-3700, HITACHI Ltd., Japan) at 10 kV. The samples were covered with platinum to perform these measurements. The crystal structure was examined using an X-ray diffractometer (XRD; X’Pert PRO, PANalytical, USA) by using Cu Kα radiation (*λ* = 1.5406 Å). Patterns were collected in a 2*θ* interval of 20°–80° with increments of 0.02° (2*θ*). The resistivity of Cu films was measured using a four-point probe system (Mitsubishi Chemical Corporation, Loresta-GP MCP-T6 00, Japan). The surface composition of the Cu films was analyzed by X-ray photoelectron spectroscopy (XPS; PHI 5000 VersaProbe™, Ulvac-PHI). High-resolution XPS conditions were fixed to a constant analysis energy mode, with 58.7 eV of pass energy and a monochromatic Al source. The adhesive strength between the aerosol-deposited Cu film and the Al_2_O_3_ substrate was measured using a universal testing machine (DUT-300CM, Daekyung engineering Corp., Korea) at a loading speed of 5 mm/min. A force-displacement curve was derived from the tensile test, and the adhesive strength *F* of the Cu coatings was estimated, using the following equation; *F* = |*f*_max_|/A, where *f*_max_ is the measured peak load value of the breaking force and A is the area that was peeled off the Cu film.

## Results and Discussion

### Substrate Dependency on Properties of Cu Films

**Fig. 1:**
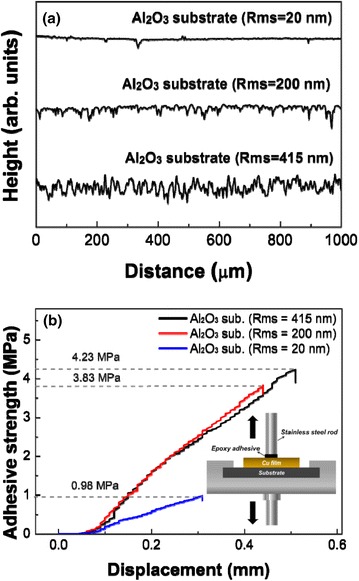
**a** Surface roughness of Al_2_O_3_ substrates and (**b**) adhesive strength of Cu films grown on Al_2_O_3_ substrates with different surface roughness values. The *inset* shows a schematic diagram of adhesion force measuring method. The adhesive strength is calculated from the maximum tensile force or pull-off force

Generally, ceramic particles with high hardness during the AD process can form the anchoring layers on metal and glass substrate with relatively low hardness [23]. However, with metallic particles that have a ductile property, it is difficult to form the anchoring layer on the hard ceramic substrate. Therefore, the bonding between metallic particles and the ceramic substrate is determined by the mechanical interlocking [20], and thereby the substrate roughness can considerably affect the adhesion strength. Figure 1a, b shows the surface roughness of the Al_2_O_3_ substrate with different root-mean-square (Rms) roughness values and the adhesive strength of Cu films depending on the substrate roughness, respectively. The inset in Fig. 1b shows a schematic diagram of the adhesive strength measuring method. First, the specimens were immobilized in a designed support, and a cylindrical rod (10 mm diameter, 50 mm length) or square rod (10 mm diameter, 50 mm length) was then bonded to the surface of the Cu film using epoxy adhesive. The rod was then pulled out until the Cu coatings peeled off, using a universal testing machine at a loading speed of 5 mm/min. As shown in Fig. 1b, the Cu films formed on the flat Al_2_O_3_ substrate (Rms = 20 nm) have very low adhesive strength (0.98 MPa) and low deposition rate (~0.1 μm/min) because it is difficult to form the mechanical interlocking. As the Rms roughness of the Al_2_O_3_ substrate increases from 200 to 415 nm, the adhesive strength increases from 3.83 to 4.23 MPa, respectively. This means that the rough surface of the Al_2_O_3_ substrate can give strong adhesion strength compared to the flat Al_2_O_3_ substrate. Moreover, the deposition rate of Cu films was increased from 5 to 8 μm/min when using a rough Al_2_O_3_ substrate. Therefore, the rough Al_2_O_3_ substrates are required to improve the deposition rate and the adhesive strength.

**Fig. 2:**
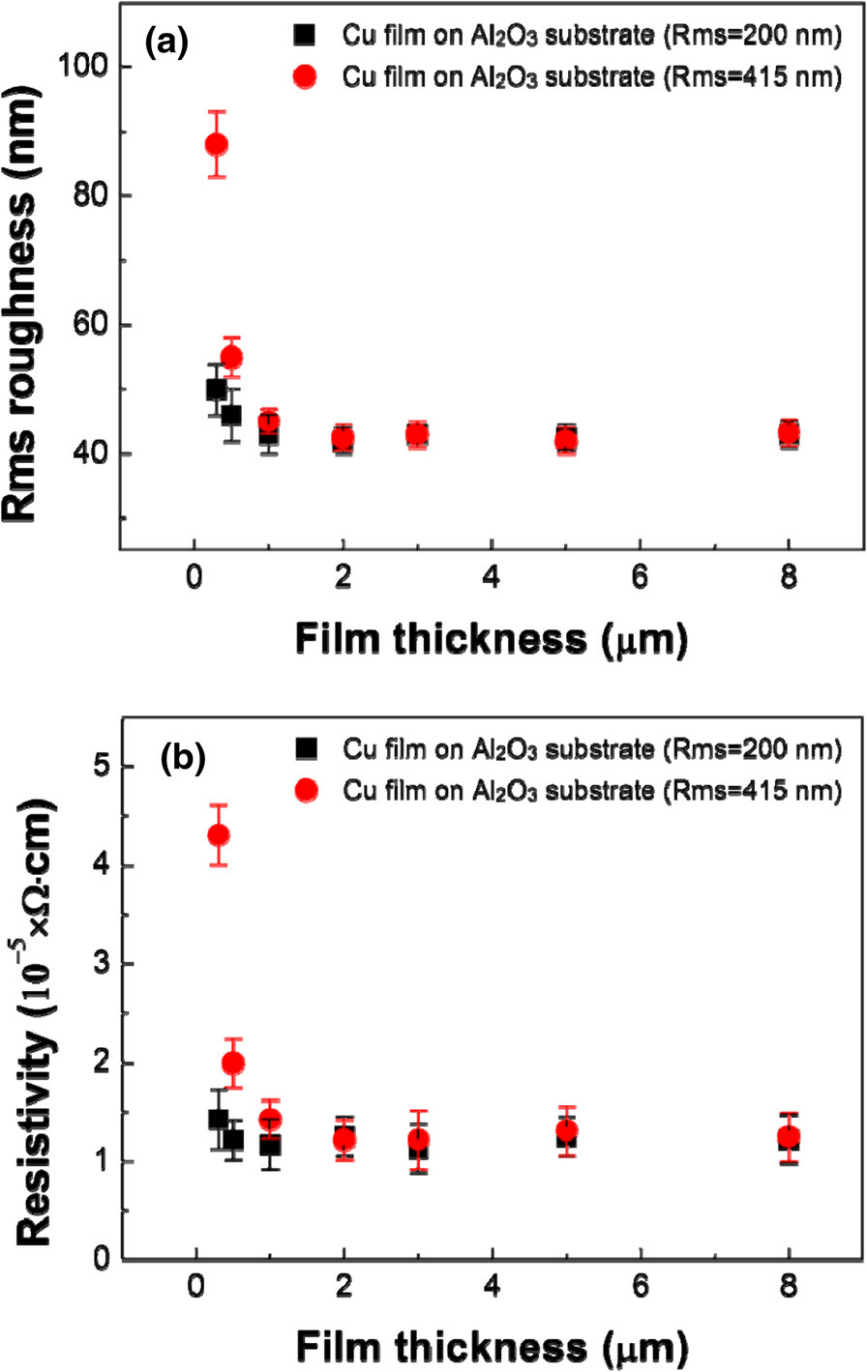
**a** Surface roughness and (**b**) resistivity of Cu films grown on Al_2_O_3_ substrates, with different surface roughness values as a function of the film thickness

Figure 2 shows the surface roughness and the resistivity of Cu films deposited on Al_2_O_3_ substrates as a function of film thickness. The resistivity and surface roughness of Cu films rapidly increased at the initial stage because the Cu particles partially filled the rough surface of the Al_2_O_3_ substrate. The resistivity and surface roughness of Cu films have a saturating trend with the growth of Cu films. The resistivity of Cu films ranged from 9. 2 to 13.5 μΩ cm. These results indicate that a porous Cu film at the initial stages has a considerable influence on the increase in the resistivity at early stage growth process. In consideration of the above results, rough Al_2_O_3_ substrate (Rms = 415 nm) has been chosen as the substrate for the deposition of Cu films.

### Growth Process of Cu Films

**Fig. 3:**
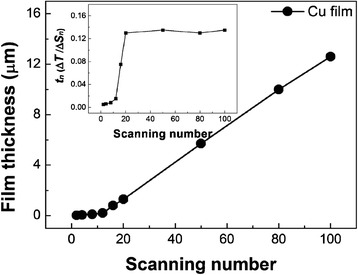
Thickness of Cu films grown on Al_2_O_3_ substrates as a function of the scanning number. The inset shows the correlation between *t*_n_(Δ*T*/Δ*S*_*n*_) and the scanning number, where Δ*T* is the variation of the film’s thickness, Δ*S*_*n*_ is the variation of the scanning number, and *n* is the scanning number

To research the AD-induced growth of the Cu films, the film growth processes, from the initial stages to the final thick films, were investigated. Figure 3 shows the change of film thickness on the scanning number that ranges from 2 scans to 100 scans. Cu films deposited on Al_2_O_3_ substrate increased nonlinearly and had very low thickness within the scan range from 1 scan to 16 scans. After 20 scans, the thickness of the Cu films increased linearly. These phenomena imply that it is difficult to form coating layers in the initial stages. To support these results, the variation of film thickness for every scan (*t*_n_) is represented as follows: *t*_n_ = ∆*T*/∆*S*_*n*_. *t*_(2–16)_ for the initial stages had very low values, but *t*_(20–100)_ after 20 scans shows a saturating trend. From the low value of *t*_(2–16)_, it can be inferred that Cu particles filled the rough surface of the Al_2_O_3_ substrate.

**Fig. 4:**
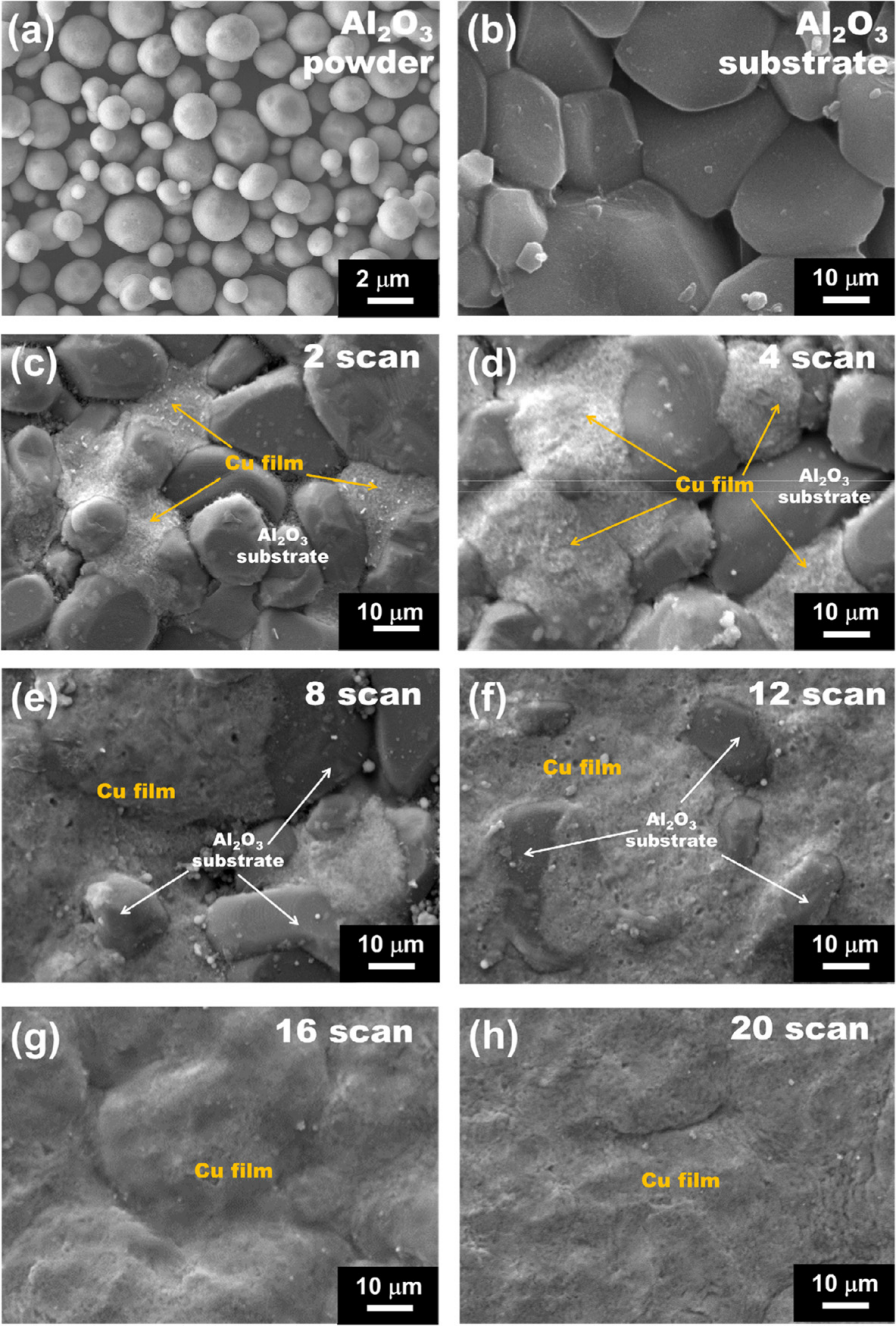
SEM micrograph of (**a**) Cu powders and (**b**) surface of Al_2_O_3_ substrates. SEM micrographs of Cu films deposited on rough Al_2_O_3_ substrates with increasing scans: (**c**) 2, (**d**) 4, (**e**) 8, (**f**) 12, (**g**) 16, and (**h**) 20 scans

**Fig. 5:**
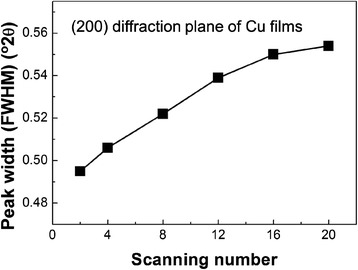
Peak width (at FWHM) of the Cu (200) plane as a function of the number of scans

To explain the causes of these phenomena, the film growth in the initial stages was investigated by observing the microstructure. The aerosol concentration was reduced, and the Cu films were scanned on the rough Al_2_O_3_ substrate in steps. Figure 4 shows a series of microstructures of Cu films that were observed with an increased number of scans. Many flattened Cu particles partially filled the rough surface of the Al_2_O_3_ substrate after two scans. As the scans increase from 4 to 16, Cu particles become significantly flattened and progressively fill the rough surface of the Al_2_O_3_ substrate. This means that the mechanical interlocking between the Al_2_O_3_ substrate and Cu particles and the plastic deformation of the Cu particles during the AD process play an important role in the formation of bonding between the substrate and the particles. After 20 scans, Cu films with a dense structure are observed as shown in Fig. 4f. These observed microstructural results are supported by XRD measurements. Figure 5 shows the peak width (at FWHM) of (200) diffraction plane of Cu films as a function of the number of scans. As the number of scans increases, the FWHM of Cu films increases. These results indicate that the growth of Cu films is significantly associated with the plastic deformation due to the collision of impacted particles. Moreover, the mechanical interlocking between Cu particles and the Al_2_O_3_ substrate in the initial stages plays important roles in making Cu films with dense structures.

### Cu Films Grown on Al_2_O_3_ Substrates

**Fig. 6:**
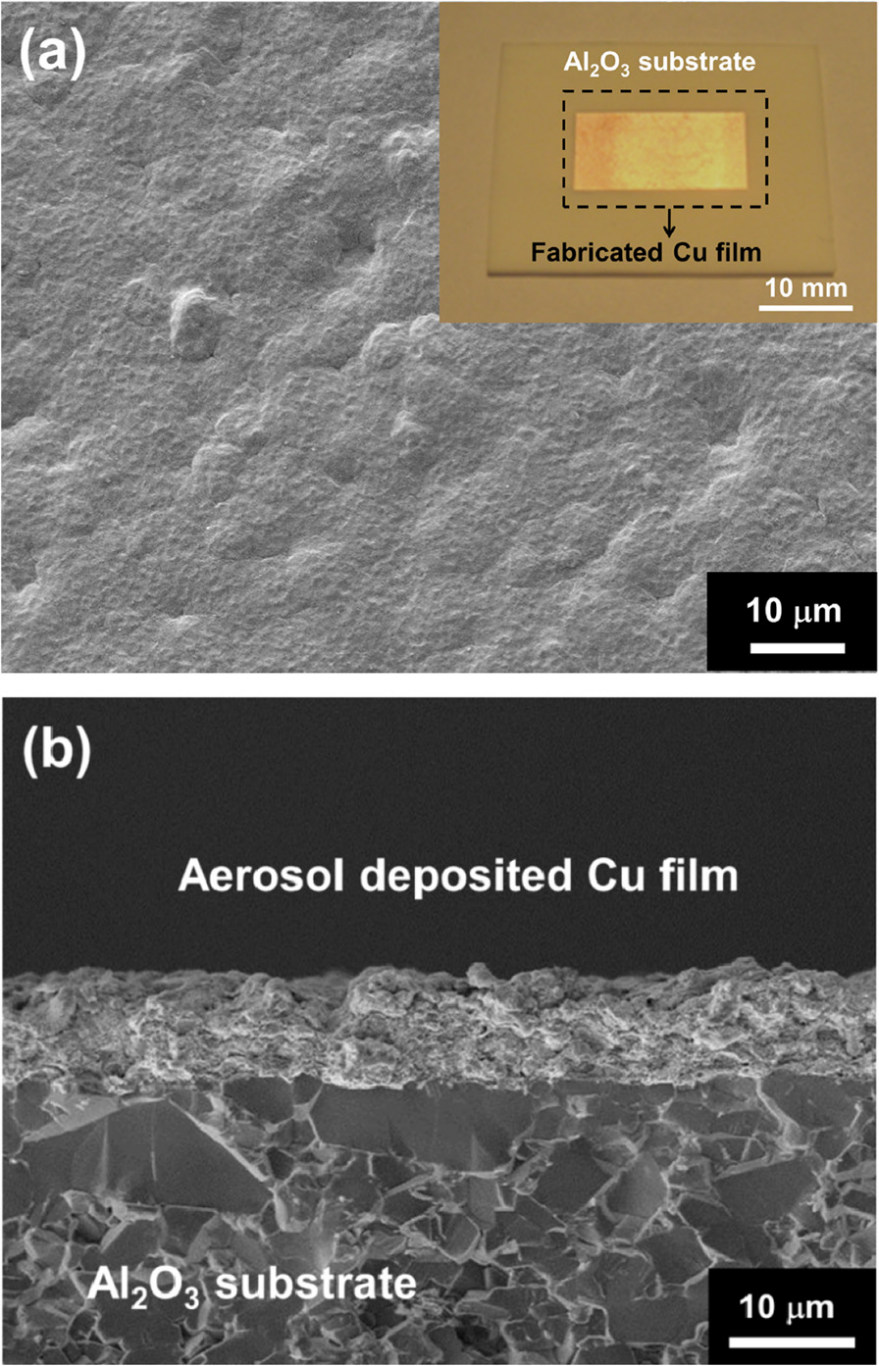
SEM micrographs of Cu films grown on Al_2_O_3_ substrates at room temperature: (**a**) plane-view SEM image and (**b**) cross-sectional SEM image. The *inset* shows the appearance of aerosol-deposited Cu films on the Al_2_O_3_ substrate

**Fig. 7:**
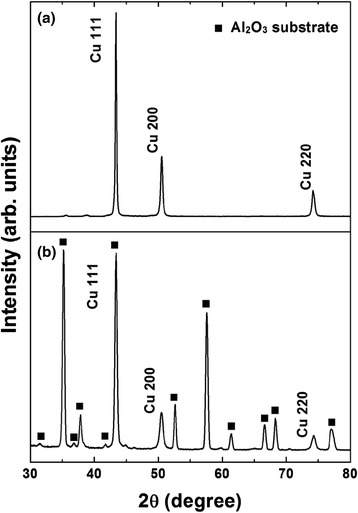
XRD peak patterns of the Cu films deposited by AD: (**a**) Cu powder and (**b**) fabricated Cu films on Al_2_O_3_ substrates

Cu films were fabricated on Al_2_O_3_ substrates with high deposition rates at room temperature. Figure 6a, b shows plane-view and cross-sectional SEM images, respectively. The film exhibited a dense microstructure with surface roughness in the range of 15–30 nm. The cross section of a Cu film shows a film thickness of approximately 8 μm, and very few pores were observed in the Cu films. Figure 7 shows the X-ray diffraction (XRD) patterns of Cu powder and an aerosol-deposited Cu film on α-Al_2_O_3_ polycrystalline substrates. The spectra of the deposited Cu films showed broader peaks and high full width at half maximum (FWHM) in comparison with that of the Cu powders. It is well known that peak broadening can be caused by both a reduction in crystallite size and an increase in lattice strain [24]. In the AD process, the peak broadening of ceramic films is mainly influenced by the reduction in crystallite size [17, 19, 25–28]. However, it is believed that the peak broadening of Cu films is mainly dependent on the internal strain, because metal materials have ductility [20]. The XRD analysis and observation of the microstructure indicate that Cu films with a dense structure are formed by plastic deformation of Cu particles due to the hammering effect.

**Fig. 8:**
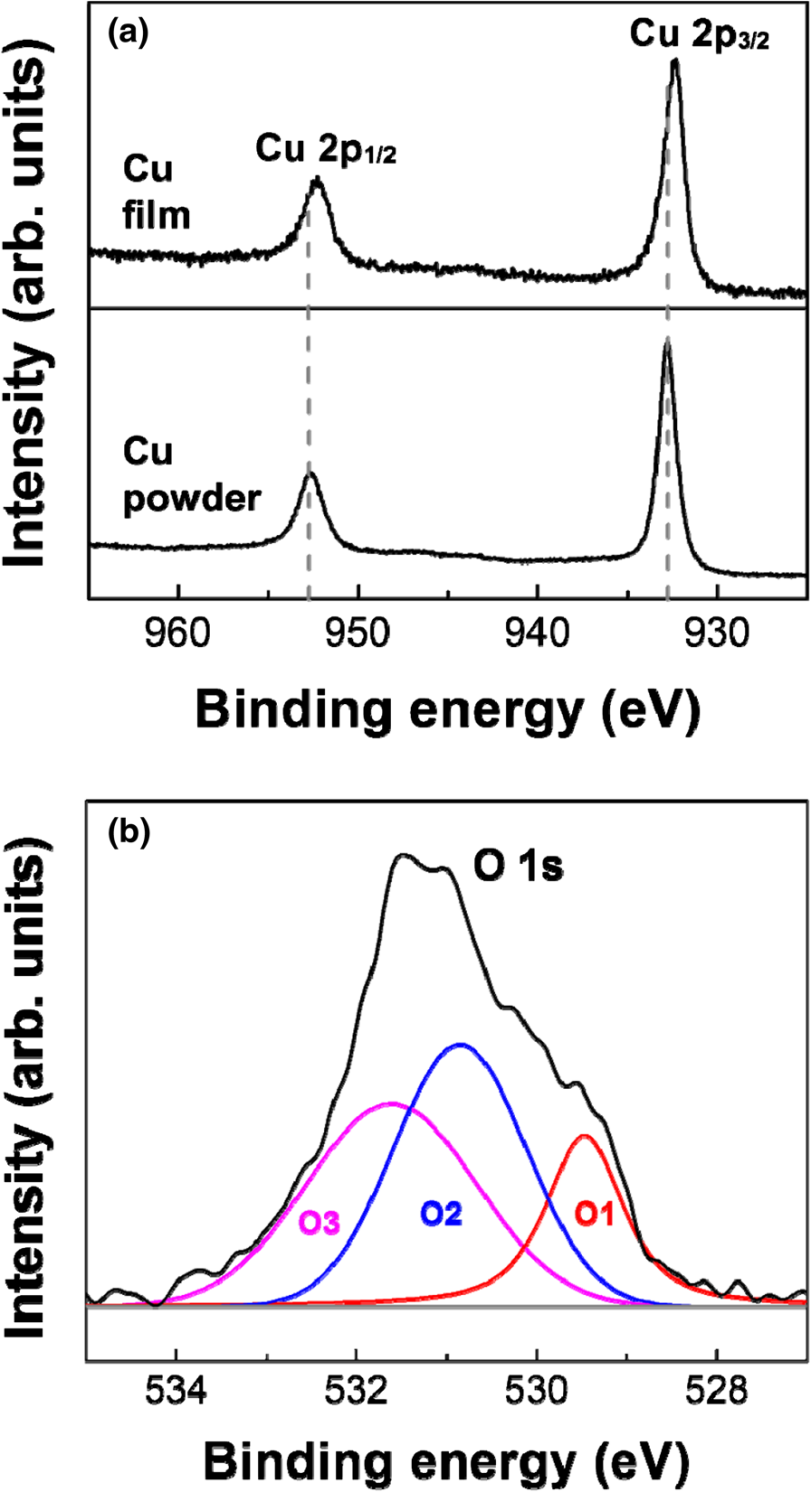
Comparison of the XPS core-level spectra of the Cu powder and Cu films deposited by AD. (**a**) Cu 2p peak of Cu powder and Cu films and (**b**) deconvoluted O 1s peak of the films

Figure 8a shows the XPS profiles corresponding to the Cu 2p spectrum region of the Cu powders and Cu films. Two peaks of the Cu powder, observed binding energy 952.5, and 932.7 eV, corresponding to the Cu 2p_1/2_ and Cu 2p_3/2_ spectrum regions, respectively, were assigned to the pure metallic Cu peak [29, 30]. However, the peaks of Cu films had shifted toward a slightly lower binding energy of 952.3 and 932.5 eV, respectively. It was thought that the chemical shift of Cu 2p is related to the Cu oxide phases generated due to the collision of Cu particles. The O 1s peak of the Cu films was also analyzed to identify the Cu oxide phases, as shown in Fig. 8b, and could be fitted by three nearly Gaussian components (O1, O2, and O3). The highest binding energy component (O3) centered at 531.6 eV (peak area = 39.68 %) is ascribed to adsorbed hydroxide on the surface of Cu films [31]. The features at ~530.8 eV (peak area = 39.87 %) and ~529.5 eV (peak area = 20.45 %) can be ascribed to the Cu_2_O and CuO species [29, 30]. In previous research, a numerical simulation of a single particle impact in AD showed that the maximum temperature was 500–600 K. After impact between particles, the maximum temperature at the interface of two particles was about 1250 K. This means that some of the interfaces of the Cu particles can be oxidized to CuO or Cu_2_O due to the high temperature after the collision between Cu particles. For this reason, the resistivity of Cu films ranged from 9.2 to 12.5 μΩ cm, which was approximately five to seven times larger than that of bulk copper (1.67 μΩ cm) [7, 32]. It is thought that the increase of the resistivity is attributed to the OH adsorption and the formation of the CuO or Cu_2_O species.

**Fig. 9:**
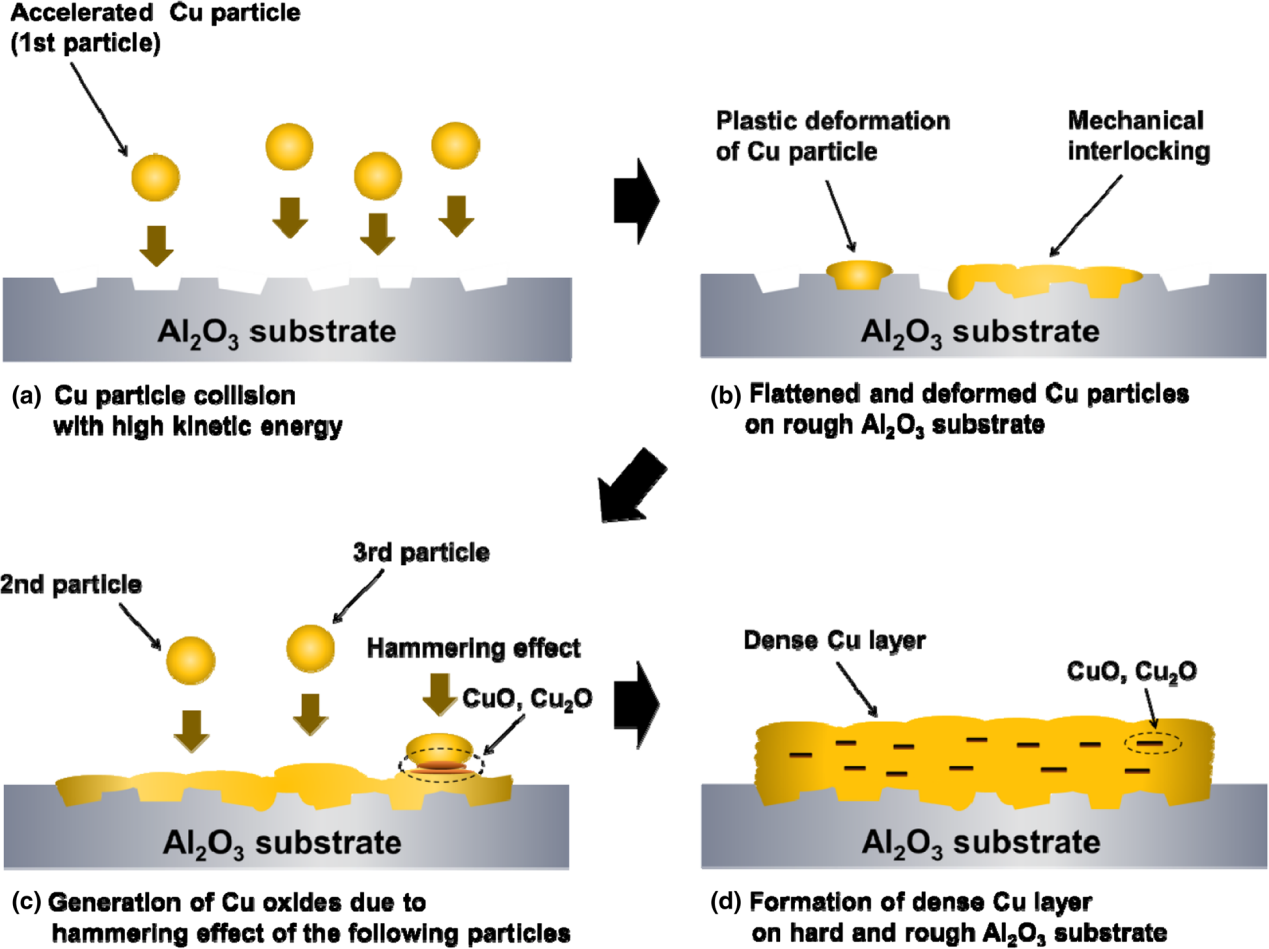
Schematic diagram of initial mechanical interlocking on Al_2_O_3_ substrates and the growth of Cu films by the AD process. (**a**) Cu particle collision with high kinetic energy. (**b**) Flattened and deformed Cu particles on rough Al2O3 substrate. (**c**) Generation of Cu oxides due to hammering effect of the following particles. (**d**) Formation of dense Cu layer on hard and rough Al_2_O_3_ substrate

Based on these results, the growth of the Cu films on the Al_2_O_3_ substrates is depicted in Fig. 9. The Cu particles accelerated by gas flow directly collide with the rough Al_2_O_3_ substrates. The impacted particles are flattened and deformed and fill the rough Al_2_O_3_ substrate. The following Cu particles are continuously impacted on the previously deposited particles. The hammering effect of subsequent particles makes the Cu films denser and causes plastic deformation and heat energy of the particle even at room temperature. During the above process, the boundaries between each deposited Cu particle can form Cu oxides (Cu_2_O and CuO) due to the elevated temperature caused by the kinetic energy of the impacted particles. As a result, the Cu films have dense surface morphologies due to the plastic deformation of impacted particles.

In this research, we confirmed that the AD process, with merits such as high deposition rate, capability for room-temperature processing, and low resistivity, can be used as a metallization process for microwave devices. Moreover, it was explained that the growth of Cu films was closely related with the plastic deformation of particles and the mechanical interlocking between particles and rough substrate. In a future work, we expect to investigate the complementary electrical properties of Cu films through the pre-treatment process of powders and the post-annealing process of coating layers.

## Conclusions

Dense Cu films with a thickness of ~8 μm were deposited on Al_2_O_3_ substrates at room temperature using an AD process. The Cu films had a low electrical resistivity of 9.2–12.5 μΩ cm, but this was five to seven times larger than that of bulk copper. The causes of the increase in the resistivity were explained through XPS analysis. It was revealed that the generation of Cu_2_O and CuO oxides in Cu films affects the electrical resistivity. Based on the XRD analysis, it was also explained that the increase in FWHM of Cu films is due to the internal strain during the collision of Cu particles. To observe the particle behaviors when Cu particles move to the Al_2_O_3_ substrates, the initial growth stage of Cu films were investigated in steps. It was confirmed that the bonding between Al_2_O_3_ substrates and Cu particles strongly depends on the plastic deformation and mechanical interlocking. Moreover, the effect of substrate roughness on the resistivity, the adhesive strength, and the surface roughness of Cu films were investigated to understand the mechanical interlocking between Al_2_O_3_ substrates and Cu particles. The rough Al_2_O_3_ substrates showed a superior adhesive strength to the smooth Al_2_O_3_ substrates.
